# The Role of Portal Vein Thrombosis in the Clinical Course of Inflammatory Bowel Diseases: Report on Three Cases and Review of the Literature

**DOI:** 10.1155/2012/916428

**Published:** 2012-10-11

**Authors:** Emanuele Sinagra, Emma Aragona, Claudia Romano, Simonetta Maisano, Ambrogio Orlando, Roberto Virdone, Lorenzo Tesè, Irene Modesto, Valeria Criscuoli, Mario Cottone

**Affiliations:** ^1^Division of Internal Medicine, DIBIMIS, Ospedali Riuniti Villa Sofia-Vincenzo Cervello, Via Trabucco 180, 90100 Palermo, Italy; ^2^Division of Radiology, Ospedali Riuniti Villa Sofia-Vincenzo Cervello, Via Trabucco 180, 90100 Palermo, Italy

## Abstract

Inflammatory bowel diseases are associated with an increased risk of vascular complications. The most important are arterial and venous thromboembolisms, which are considered as specific extraintestinal manifestations of inflammatory bowel diseases. Among venous thromboembolism events, portal vein thrombosis has been described in inflammatory bowel diseases. We report three cases of portal vein thrombosis occurring in patients with active inflammatory bowel disease. In two of them, hepatic abscess was present. Furthermore, we performed a systematic review based on the clinical literature published on this topic.

## 1. Introduction

In inflammatory bowel diseases (IBD) there is a high incidence of thromboembolic complications, as a consequence of hypercoagulability status. Thrombosis of the splenic-mesenteric-portal system is a rare event; however, due to advances in diagnostic tools, many cases of mesenteric or portal vein thrombosis have recently been described in IBD patients; its incidence seems to be higher than among the general population [1]. First of all we report three cases of portal vein thrombosis in IBD patients; afterwards, we assess the epidemiology, the clinical course, the diagnosis, and the therapeutic approach of this complication of IBD, performing a systematic review based on the clinical literature.

An in-depth search in PubMed was based on four search terms: “inflammatory bowel diseases” OR “Crohn's disease” OR “ulcerative colitis” AND “portal vein thrombosis” (MeSH terms). Review articles and case reports not regarding this topic were excluded. The remaining articles were categorised by topic and summarised. 

Table 1 summarizes risk factors, treatment, and outcome of portal vein thrombosis (PVT) in IBD patients reported in the literature.

## 2. Case  1

**Figure 1:**
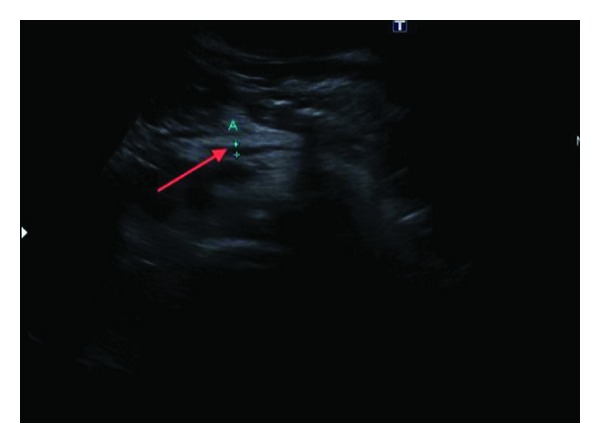
Abdominal US showing a portal vein thrombosis extended to intrahepatic branches.

**Figure 2:**
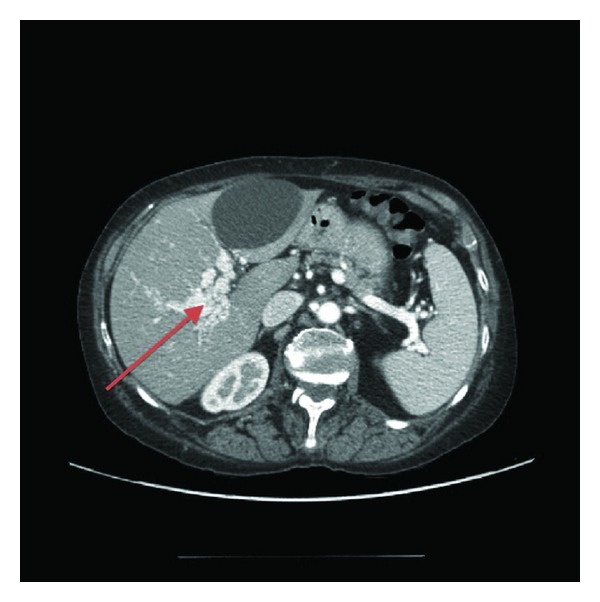
Residual portal cavernoma showed at CT scan.

A 66-year-old woman, with a 10-year diagnosis of inflammatory ileal Crohn's disease (CD), on treatment with budesonide for active disease, was admitted to our unit for fever with chills. There was no history of previous thromboembolic events. Physical examination revealed tachycardia, without any abdominal tenderness or masses; blood tests showed leucocytosis (13000 white blood cells (WBC)/mm^3^), thrombocytosis (360000 platelets/mm^3^), and increased ESR (25 mm/Ih) and PCR (18 mg%); 3 blood cultures isolated *Gemella Haemolysans* and *Sphingomonas paucimobilis*. Ultrasound (US) doppler and computer tomography (CT) scan showed a small abscess in liver l and PVT extending to the intrahepatic branches (Figure 1). Coagulation study did not show any abnormalities (antithrombin, protein C and S activity, plasma homocysteine level, PT, and aPTT were normal) and no mutation was detected in factor II, V, MTHFR, and PAI-I genes; antibody anticardiolipin and antiphospholipid antibodies were negative; no mutation of JAK-2 gene was found. The patient was treated with low-molecular-weight heparin (LMWH) (1 mg/kg/twice daily) and imipenem on the basis of the blood culture results for 3 weeks and then amoxicillin/clavulanic acid for further 2 weeks on the basis of the results of blood culture and relative sensibility testing, with disappearance of fever. A further US Doppler, performed one month after, showed a complete patency of portal three and disappearance of hepatic abscesses. LMWH was suspended after 3 months. At the last outpatient control, the patient had optimal clinical and nutritional status, while US Doppler showed a residual portal cavernoma, confirmed at CT scan (Figure 2).

## 3. Case  2

A 41-year-old woman with a family history of IBD was admitted to our unit because of the onset of bloody diarrhoea. There was no history of previous tromboembolic events. Clinical examination showed fever (38.5°C), hypotension (80/50 mmHg), tachycardia (115 beats/minute), tachypnea, and abdominal distension without bowel movements; blood tests showed leucocytosis (18000 white blood cells (WBC)/mm^3^) and increased ESR (30 mm/Ih) and PCR (25 mg%). An abdominal plain X-ray showed a dilation of the transverse colon (diameter 6 cm), while an abdominal US Doppler and CT scan showed ascites, complete thrombosis of portal, mesenteric and splenic veins, and wall thickening of the large bowel. The patient received steroids, ciprofloxacin, and metronidazole. Coagulation study showed heterozygosis for MTHFR gene mutation; antithrombin, protein C and S activity, plasma homocysteine level, PT, and aPTT were normal; anticardiolipin and anti-phospholipid antibodies were negative; no mutation of the JAK-2 gene was found. During hospitalization the patient complained of acute dyspnoea, with oxygen desaturation. A chest CT scan showed pulmonary embolism. Treatment with LMWH (1 mg/kg/twice daily) was started, with progressive disappearance of respiratory complaints. Colonoscopy with biopsy, performed after resolution of the colon dilation, diagnosed an ulcerative pancolitis. The patient continued treatment with steroids at tapering doses and started azathioprine. A US Doppler after 1 month showed complete recanalization of portal three. LMWH was discontinued after 3 months. A US Doppler control after 6 months showed patent portal tree.

## 4. Case  3

**Figure 3:**
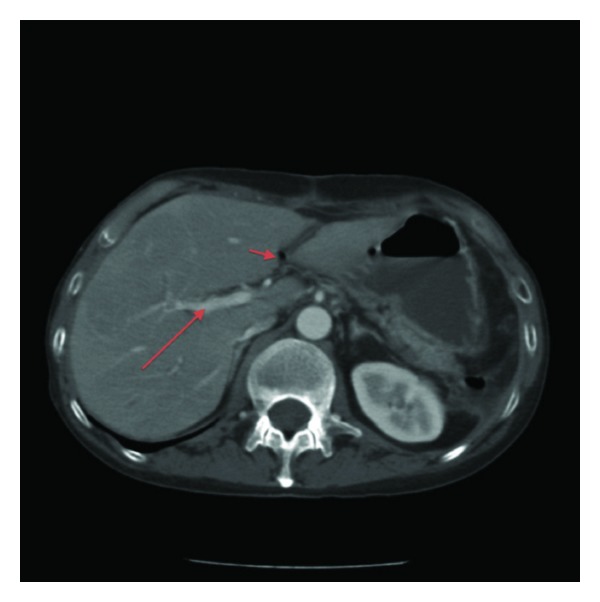
CT scan showing gas in portal vein and a liver abscess.

A 52-year-old male with a 20-year history of small bowel CD resected twice, after recent detection of stenotic recurrence, was admitted to our unit because of the onset of fever and diarrhoea. No history of previous thromboembolic events was present. Clinical examination showed fever (39°C) and painful abdominal distension; blood tests showed leucocytosis (16000 white blood cells (WBC)/mm^3^) and increased ESR (22 mm/Ih) and PCR (24 mg%). A CT scan showed gas in the portal vein and a liver abscess (Figure 3). Coagulation study (antithrombin, protein C and S activity, plasma homocysteine level, PT, and aPTT) did not show abnormalities and no mutations were detected in factor II, V, MTHFR, and PAI-I genes; anticardiolipin and antiphospholipid antibodies were negative; no mutation of JAK-2 gene was found. The patient was treated with ciprofloxacin and metronidazole and started LMWH (1 mg/kg/twice daily). After 4 weeks a CT scan showed patent portal and mesenteric vein. After drainage of abdominal abscesses, a surgical operation was performed for treatment of strictures and fistula. LMWH was stopped after three months. To date the patient is asymptomatic, with normal and patent portal vein detected with periodic US controls.

## 5. Discussion

These three cases of portal thrombosis in IBD show that this complication, despite its rarity, needs to be searched for in case of clinical suspicion, and that treatment with LMWH leads to complete resolution of this complication. 

Portal/mesenteric vein thrombosis, particularly in the nonsurgical setting, is a rare complication in IBD patients [2]. However, its incidence appears to be higher than in the general population [1], linked to the high risk of venous thromboembolism (VTE) in IBD [3–6].

Portal/mesenteric vein thrombosis in IBD, seen in a study performed by the Mayo Clinic, was reported in 1.3% of cases, with a mortality rate of 50% [7]. In our research, including case reports, case series, letters to the editors, and retrospective studies, we identified 82 cases of PVT in IBD patients (Table 1). Bruining et al. [8] analyzed the records of 357 consecutive patients with previously diagnosed CD identified with computed tomography enterography. In this series 6 patients (1.7%) had portal/mesenteric vein thrombosis. In the study by Jackson et al. [9] among 9 IBD patients, 8 subjects (4 with CD and 4 with UC) developed mesenteric venous thrombosis (5 located in the SMV and 3 located in a branch of the portal vein). The mean time to diagnosis of IBD to thrombosis was 24.6 ± 13.5 years, while 5 of the 9 patients developed mesenteric venous thrombosis while their IBD was clinically in remission.

Maconi et al. [10], in their series, identified a prevalence of 0.17%, with a mean time from the diagnosis of IBD to the detection of IBD of 14.8 ± 6.6 years; in 4 patients the diagnosis of PVT was made while their IBD (CD) was in clinical remission, while 4 patients (2 UC patients and 2 CD patients) had an active disease.

Portal thrombosis occurs more frequently in the setting of abdominal surgery [1]. Fichera et al. [11] reported a 4.8% incidence of superior mesenteric vein (SMV) thrombosis among 83 patients undergoing colectomy for IBD.

PVT has been seen in UC patients following restorative proctocolectomy [12–14]. In a retrospective study, PVT was found in up to 45% of CT scans done after ileal pouch-anal anastomosis (IPAA) for UC [12]. 

Portal/mesenteric vein thrombosis, and more generally VTE, has been considered a manifestation which seems to be related to intestinal inflammatory activity. Indeed, in a different chronic inflammatory disease like Rheumatoid Arthritis, in which there is no intestinal inflammation, there is no increased incidence of PVT compared to the general population [15]. However, IBD patients in remission also have an increased risk of VTE [3]. Hence additional factors, other than inflammation, are probably involved [16].

The causes of portal/mesenteric thrombosis in IBD are manifold; in most patients, recognized acquired prothrombotic factors can be identified, such as inflammation, immobilization, extent of colon disease, surgery, central catheters, corticosteroids, and smoking [17–19].

On the other hand, thromboembolic complications in IBD, such as PVT, may be associated with coagulation abnormalities, which are induced by chronic bowel inflammation [15, 20]. Patients with IBD have increased platelet counts, factor V and VIII levels, and fibrinogen levels and decreased antithrombin III levels, all of which can increase the risk of thrombosis [2, 20–26]. 

Several kinds of presentation of portal/mesenteric vein thrombosis have been reported: IBD flares and sepsis (especially perioperative) have been more frequently described; however, other rare modalities reported are variceal bleeding (VB) and hepatic portal venous gas (HVPG) [27–32]. 

Thromboembolic complications of IBD are by no means benign: the mortality rate has been reported to be as high as 22%–25% [4, 7, 18]. As mentioned above, they can be manifested at the onset of IBD, either during an IBD flare or when the disease is in remission. 

The most widely recognized pathophysiological factor of PVT is the presence of ulceration and the loss of integrity of the normal mucosa barrier in the bowel, which may result in microbial invasion or translocation of the portal vein system, with seeding in the parenchyma giving rise to portal pylephlebitis—defined as septic thrombophlebitis of the portal vein or of its tributaries—and PVT. 

However, the presence of portal venous gas (PVG) associated with PVT could be a rare but serious, even catastrophic condition in IBD patients [33]; to date 21 cases of PVG associated with CD have been reported in the scientific literature [34–37]. Among 182 case studies reported by Chande et al. [38], patients with UC or CD comprised 4% of the total. 

PVG in IBD patients can be caused by mucosal damage alone, or it can occur in combination with bowel distension, sepsis, and invasion by gas-producing bacteria, or after colonoscopy, upper gastrointestinal barium examination, barium enema, or blunt abdominal trauma. PVG is not always a surgical condition, and its treatment should be based on the underlying disease and the patient's current clinical condition [35]. Although PVG itself is not a prognostic indicator, PVG combined with pylephlebitis can be regarded as an indicator of poor prognosis [35].

Regarding diagnosis of PVT in IBD patients, abdominal US with colour Doppler proves crucial. However, CT scan is more sensitive than US (which is more operator dependent and gives results that are less reproducible than those of CT) for detecting a thrombus within the splenic and mesenteric veins, and therefore it should be the preferred imaging technique for detecting both thrombi and pericolonic abscesses, especially in a setting of pylephlebitis [38]. A CT scan also provides a better assessment of bowel viability and the presence of a perforation, thus allowing one to work better in selecting patients for conservative management [39]. 

Anticoagulants, such as LMWH and warfarin, are mainstays of primary therapy, even in the setting of gastrointestinal bleeding [17]. The duration of systemic anticoagulation is not well established in the literature [40]. In the presence of a congenital hypercoagulable state, consideration should be given to lifelong systemic anticoagulation, although in other prothrombotic conditions, a six-month course provides adequate coverage. The use of anticoagulants in secondary prevention is recommended but can be limited by continued bleeding [41]. LMWH and devices such as inferior vena cava filters have been used with success, according to the literature [17, 38]. Both unfractionated heparin and LMWH have also been studied for induction of remission in UC without thromboembolic complications, but they have shown no benefit over standard therapies alone [42]. However, according to a recent Cochrane systematic review and meta-analysis [38] there is evidence to suggest that LMWH may be effective for the treatment of active UC; when administered by extended colon-release tablets, LMWH was more effective than placebo for treating outpatients with mild to moderate disease. This benefit needs to be confirmed by further randomized controlled trials. The same benefits were not seen when LMWH was administered subcutaneously at lower doses, while there is no evidence to support the use of unfractionated heparin for the treatment of UC.

Other therapeutic approaches for acute portal/mesenteric vein thrombosis are, in extreme cases, surgical interventions, thrombolysis, and intravascular thrombectomy devices [43–51].

Minimizing modifiable risk factors is also a mainstay of therapy. Smoking, oral contraceptives, and hormone replacement therapy should be discontinued, and prolonged immobility should be avoided as far as possible [17]. 

Summing up, whether or not to anticoagulate patients with IBD and PVT remains controversial: certainly, anticoagulation in the setting of active IBD may result in increased haemorrhage risk. Those patients whose portal/mesenteric vein thrombosis developed in the setting of significant systemic inflammation should be treated on an individual basis [14], just as preoperative prophylaxis in IBD patients undergoing abdominal surgery should be further clarified.

In conclusion, portal/mesenteric vein thrombosis proves to be more frequent in IBD patients than in healthy controls, and among IBD patients this complication occurs more frequently in the setting of abdominal surgery. Several factors, inherited or acquired, related or not to IBD, seem to be involved in its pathogenesis. Abdominal US is the first-line technique to detect portal/mesenteric vein thrombosis, though, to date, CT scan remains the gold standard technique. To date, there is no proved effective treatment for pylephlebitis and for portal/mesenteric vein thrombosis in IBD, as the natural history of these conditions is not well defined. However, in IBD patients, especially ones with bloody diarrhoea, there is still a controversy as to whether anticoagulation should be maintained lifelong, just as there is insufficient information about the timing and duration of treatment with anticoagulation in the subset of IBD patients without known coagulation disorders. 

## Figures and Tables

**Table 1:** Characteristics of the reports of the literature regarding the portal vein thrombosis in inflammatory bowel disease.

Author	No. of patients (IBD)	Biochemical risk factors for PVT	Therapy	Outcome
Maconi	8 (6 CD; 2 UC)	(i) Lupus anticoagulant (1) (ii) Increased von Willebrand factor (2) (iii) Increased homocysteine levels (4 with mutation of MTHFR-677-3 Ht)	Anticoagulant (4)	Recanalization
Lefevre	1 (UC)	Ht for prothrombin-G20210A mutation	Oral anticoagulant	Recanalization
Jackson	3 (1 UC; 2 CD)	Lupus anticoagulant + Ht for factor V Leiden mutation (1 pt)	Oral anticoagulant	NA
Ibele	1 (UC)	None	Oral anticoagulant	Recanalization
Di Fabio	1 (CD)	None	Thrombobectomy + oral anticoagulant	Recanalization
Aguas	1 (CD)	None	Heparin	Recanalization
Palkovist	1 (UD)	None	LMWH	Portal cavernoma
Latzman	1 (CD)	Ht for prothrombin-G20210A mutaion	Thrombobectomy + oral anticoagulant	Recanalization
Hatoum	3 (CD)	Factor V Leiden mutation (1)	LMWH + oral anticoagulant (1 pt)	Recanalization
Shaked	1 (CD)	None	Heparin + oral anticoagulant	Recanalization
Guglielmi	1 (CD)	None	Thrombolysis + LMWH	Recanalization
Verna	1 (UC)	Elevated FVII	No therapy	Atrophy of left hepatic lobe
Mijnhout	1 (CD)	1 (CD)	Heparin + oral anticoagulant	Recanalization
Fichera	4 (1 CD, 3 UC)	Ht for prothrombin-G20210A mutation	Oral anticoagulant	Recanalization
Remzi	41 (26 UC, 15 IC)	NA	Anticoagulation (8 pts)	Recanalization (5/13 pts) Portal cavernoma (1/13 pt) No change (7/13 pts)
Hagimoto	1 (UC)	NA	Thrombolysis + oral anticoagulant	Recanalization
Schafer	1 (CD)	None	Thrombolysis	Recanalization
Farkas	1 (UC)	None	Oral anticoagulant	Recanalization
Tsujikawa	1 (CD)	None	Thrombolysis + heparin + oral anticoagulant	Recanalization
Tung	1 (CD)	None	LMWH	Recanalization
Miyazaki	1 (UC)	None	Thrombolysis + oral anticoagulant	Recanalization
Irving	4 (CD)	None	LMWH + oral anticoagulant	Recanalization
Mathieu	1 (CD)	Acquired protein C deficiency	LMWH	Recanalization
Crowe	1 (CD)	None	Oral anticoagulant	Recanalization
Brinberg	1 (CD)	None	Heparin	Recanalization

Ht: heterozygosis, LMWH: low molecular weight heparin, IC: indeterminate colitis, UC: ulcerative colitis, CD: Crohn's disease, and MTHFR: methyl-tetrahydrofolate reductase.
